# Combined perceptual and chemical analyses show no compelling evidence for ovulatory cycle shifts in women’s axillary odour

**DOI:** 10.1098/rspb.2023.2712

**Published:** 2024-07-24

**Authors:** Madita Zetzsche, Brigitte M. Weiß, Marlen Kücklich, Julia Stern, Claudia Birkemeyer, Anja Widdig, Lars Penke

**Affiliations:** ^1^ Behavioural Ecology Research Group, Institute of Biology, Leipzig University, Talstraße 33, Leipzig 04103, Germany; ^2^ Department of Human Behavior, Ecology and Culture, Max Planck Institute for Evolutionary Anthropology, Deutscher Platz 6, Leipzig 04103, Germany; ^3^ Personality Psychology and Psychological Assessment, Department of Psychology, University of Bremen, Grazer Straße 2c, Bremen 28359, Germany; ^4^ Research Group of Mass Spectrometry, Institute of Analytical Chemistry, Leipzig University, Linnéstraße 3, Leipzig 04103, Germany; ^5^ Biological Personality Psychology, Georg Elias Müller Institute of Psychology, Georg August University Göttingen, Goßlerstraße 14, Göttingen 37073, Germany; ^6^ Leibniz ScienceCampus Primate Cognition, Göttingen 37073, Germany

**Keywords:** physical attractiveness, body odour, chemical fertility cues, volatile analysis, steroid hormones, gas chromatography–mass spectrometry

## Abstract

Although men’s attraction to women’s body odour has been suggested to vary over the ovulatory cycle, peaking around the fertile window, we still lack methodologically robust evidence corroborating this effect. Further, the chemical underpinnings of male preference for the odour of ovulating women remain unknown. Here, we combined perceptual and chemical analyses to investigate the axillary odour of naturally cycling women over 10 days, covering the gradual change in fertility across the ovulatory cycle with a focus on fertile days. The fertile state was confirmed by urinary ovulation tests as well as salivary oestradiol and progesterone levels. Men rated the scent of unfamiliar women, resembling a first encounter. We used multivariate analyses to relate variation in both odour ratings and chemical composition to female conception probability, temporal distance to ovulation and ovarian hormone levels. Our results provide no evidence that males prefer the odour of fertile women. Furthermore, the volatile analysis indicated no link between axillary odour composition and current fertility status. Together, our results showed no convincing support for a chemical fertility cue in women’s axillary odour, questioning the presence of olfactory fertility information that is recognizable during first encounters in modern humans.

## 1. Introduction

Human mate choice decisions are informed by the quality of potential mates [1] and, thereby, largely rely on judging aspects of their physical attractiveness (reviewed in [2]). Attractiveness has been suggested to allow immediate assessment of mate quality [3] and, as such, has the potential to affect courtship decisions.

Men particularly attend to features of female physical attractiveness when evaluating a prospective sexual partner [1,4]. Even subtle changes in a woman’s physical appearance have been suggested to provide information about her current fertile state, such as facial or vocal changes (e.g. [5–7]). Similarly, subtle changes in body odour have been suggested to convey information about women’s fertility [8], as volatile odorous compounds with putative communicative functions are produced across the human body (reviewed in [9]). Physiologically, cycle-related changes in women’s physical attractiveness might have developed as a by-product of fluctuating oestradiol and progesterone levels [5,10]. Indeed, men’s attraction to these physical traits has been shown to peak when women are fertile, indicating a potential adaptation to perceive cycle-related variation in physical attractiveness as a cue of female fertility [11]. However, more recent studies with higher methodological power challenge this view as they do not replicate these effects, for example, for women’s facial [12,13] and vocal [14] attractiveness.

Drastic hormonal changes across the ovulatory cycle, accompanied by fluctuations in conception probability, provide physiological prerequisites for translation into an olfactory cue indicative of female fertility. Ovarian steroid hormones have been shown to influence axillary sebaceous glands [15,16]. Oestrogen receptors have also been detected in apocrine glands [17], although we lack direct evidence for their response to cyclic oestrogen. Given the importance of both gland types for sociochemical communication [18], axillary odour may be a physiologically relevant candidate for conveying fertility-related volatile correlates. Indeed, men rated the scent of ovulating women as more attractive and pleasant than the odours of non-ovulating women in previous studies (e.g. [8,19–21]). Fertility-related variation has also been shown for axillary odour intensity [8,20], suggesting a negative correlation between rated odour intensity and pleasantness [20]. Additionally, the attractiveness of a woman’s scent has been related to her oestradiol and progesterone levels, indicating a close link between female body odour and reproductive hormones [10,22].

Nevertheless, we need to critically revise the reliability of this evidence. For cycle-related shifts in perceptual and hormonal responses of men to women’s body odours, null effects were reported as well [22–25]. Although Mei *et al*. [22] recently replicated that axillary odour close to ovulation received higher attractiveness ratings, they also showed that men were very poor at discriminating the female’s fertile state from odour attractiveness. Women with higher mean oestradiol, on average, smelled more attractive. However, variation in a woman’s odour attractiveness across the cycle was not associated with oestradiol and progesterone [22].

Furthermore, in most studies, men assessed fertility information of one woman over her cycle, thus simulating repeated encounters with the same woman. While this familiarity can aid in the interpretation of cues [26], the ability to detect and decode female fertility in a single encounter could enable more immediate male courtship decisions. Single encounters have only been investigated for between-women effects, comparing nearly ovulating and non-ovulating women. The findings are mixed [21,27], emphasizing the need for methodologically robust studies. In fact, the majority of evidence lacks a direct assessment of female reproductive hormones, reliable hormonal confirmation of ovulation and depicts considerable inconsistencies in estimating the fertile window [28]. Furthermore, low intra-individual sample sizes across the cycle in previous studies limit the statistical power for detecting subtle cycle-related effects [29]. Finally, testing discrete cycle phases or dichotomizing into ‘fertile’ versus ‘non-fertile’ may not fully capture the gradual changes in female fertility and reproductive hormones across the ovulatory cycle [30].

Although the presumption of female body odour containing information about fertility seems to be widely held, evidence mainly comes from studies testing solely the perception of odours. Only a few early studies investigated the volatile composition of vaginal secretions, but the results on cycle-related variation of compounds are inconclusive (reviewed in [31]). Evidence regarding axillary odour remains scarce. For reconciling the mixed evidence on olfactory fertility cues, we need to systematically establish whether cyclic variation in the chemical composition of women’s body odour does, in fact, exist, and how indicative it is of fertility.

**Figure 1.**
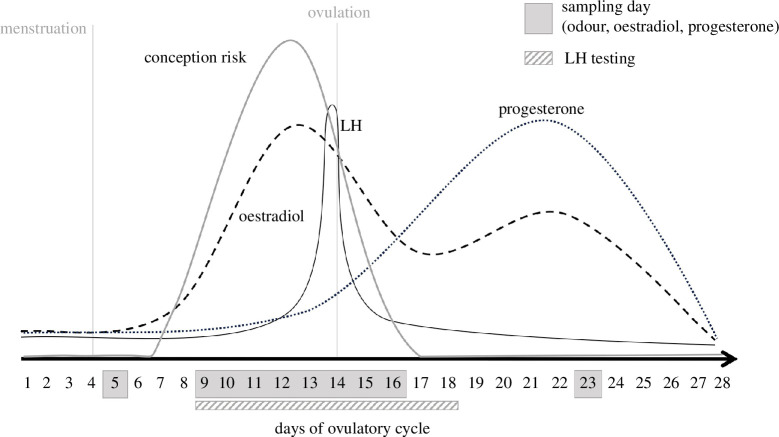
Timing of sample collection across an exemplary ovulatory cycle of 28 days. Patterns of conception risk, ovarian hormones, cycle length and timing of ovulation are idealized for illustration. Depicted are the three ovarian hormones relevant to our study with luteinizing hormone abbreviated as LH.

In the current study, women provided frequent odour samples of the armpit, as well as saliva samples and urinary ovulation tests to assess fertility. As physiological and endocrine changes across the cycle are gradual, and conception probability increases continually within the fertile window, i.e. the 5 days preceding ovulation and the day of ovulation itself [32], we expect women’s body odour to vary not only between pre-fertile, fertile and post-fertile days but also within the fertile window. To capture odour changes aligning with this fertility and endocrine gradient, we adopted a sampling design that covered pre- and post-fertile days as well as multiple days within the fertile window (figure 1). We applied test predictors for female fertility that account for different potential scenarios of odour changes around ovulation. Variation of odour composition and perception within the fertile window could align with the peak in conception risk (i.e. the probability of single unprotected intercourse resulting in conception) which occurs shortly before but changes dramatically after ovulation [33]. This scenario addresses the potential for body odour mirroring abrupt changes in female fertility during the fertile window. Also, the effect of the fluctuating levels of oestradiol and progesterone across the ovulatory cycle (‘cyclic oestradiol and progesterone’) was assessed individually, as variation in odour may correspond to variation in hormonal levels. In addition, we accounted for the possibility that humans may have evolved additional processes to inform mating decisions, with ovulation as a physiological reference point rather than solely cueing the fertile window, because anthropoid primates (including humans) are sexually active across their entire cycle, unlike most other mammals. We, therefore, further included a temporal variable in reference to the day of ovulation (distance to ovulation). An effect of this variable could shed light on hitherto unconsidered processes other than fluctuations in conception risk and reproductive hormone levels that may trigger body odour changes. For the perceptual part of the study, we aimed to investigate whether men are able to detect female fertility from a single encounter focusing on four specific predictions (i–iv). We expected that: (i) axillary odour samples of high conception risk receive higher ratings in attractiveness and pleasantness, because mating preferences are most crucial during days of high conception probability, (ii) ratings of attractiveness and pleasantness increase with lower distance to ovulation and (iii) a positive effect of oestradiol on odour ratings, as oestradiol levels drastically rise immediately preceding ovulation. Given the negative association between progesterone and the overall odour attractiveness of women reported in previous studies [10], we suppose that such negative effects may also hold at the individual cycle level. Thus, we predicted that higher progesterone levels will be associated with lower odour attractiveness ratings. Analogous to previous studies, (iv) we generally predicted an inverse effect for rated intensity for all our predictions.

Chemical profiles of women’s axillary odour, measured with gas chromatography–mass spectrometry (GC–MS), were used to assess whether changes in the abundance of chemical compounds occur in association with female fertility. We assumed chemical changes (i.e. some substances to increase or decrease) to mirror the hormonal fluctuations across the ovulatory cycle. We expected changes to be most apparent within the fertile window, indicating the imminent approach of ovulation itself.

## 2. General methods

Odour samples were collected between July 2020 and March 2021, and odour ratings were conducted between May 2021 and March 2022. Owing to the COVID-19 pandemic, strict infection protection protocols were applied (electronic supplementary material S1). Participants were supervised by a total of five women (odour collection) and three men (odour rating), all trained by the study manager.

### (a) Odour donors

We recruited naturally cycling women as odour donors, particularly targeting women who had demonstrated reliable ovulatory cycles in a previous study [34]. Eligible participants were 20–30 years old, with a regular ovulatory cycle between 21 and 35 days [35] for the last 3 months, and no hormonal contraception, pregnancy and lactation for the last 6 months. Donors had to be on a vegetarian and/or vegan diet for at least 1 month before sample collection, as meat consumption has been shown to reduce perceived body odour quality [9]. They further had to be non-smokers, have no chronic and/or hormonal illness, and have no regular use of medications or recreational drugs of any kind (see [9]). Participants were recruited via an online database at the University of Göttingen; thus, the majority of female participants represented students and academic staff members. We collected odour from 16 participants for chemical analysis. These and an additional 13 women provided odour samples for the preference tests. Thus, in total, 29 heterosexual women (aged 20–30 years, mean = 24.86, s.d. = 3.26) participated as odour donors, of which 11 women were in a romantic relationship, while 18 were single.

### (b) Sampling schedule and instructions

During an introductory meeting, the inclusion criteria were double-checked, and odour donors were instructed on the study procedure, the sampling material and infection protection measures. Participants provided demographic data and details about their ovulatory cycle (electronic supplementary material, 2.4).

Sample collection was conducted before, during and after the fertile period in 10 sessions across one ovulatory cycle per participant. For scheduling, we used the reverse cycle day method, assuming that ovulation occurs 15 days prior to the onset of subsequent menstruation (cf. [8]) and asked odour donors to the lab as follows: one session on reverse day 24 (i.e. 24 days prior to the onset of the next menses, early follicular phase), eight daily sessions on reverse days 20–13 (comprehensively covering the fertile window) and one session on reverse day 6 (mid-luteal phase, figure 1). We allowed some flexibility in scheduling to compensate for potential minor variation (up to 5 days [34]) in cycle regularity (see electronic supplementary material, S2.2). To standardize sample collection, we asked odour donors to adhere to behavioural and dietary restrictions following established procedures (cf. [8]), and women’s compliance was assessed after each session (electronic supplementary material, S2.4).

### (c) Ovulatory cycle assessment

We hormonally confirmed the day of ovulation for each woman via highly sensitive urine ovulation strips with a reported sensitivity of 10 mIU/ml (purbay® LH-tests, David One Step Ovulation Tests, Runbio Biotech, China) to measure luteinizing hormone (LH) concentrations. Participants conducted daily LH tests throughout their estimated fertile window (i.e. beginning at reverse cycle day 20). LH testing concluded with two consecutive negative tests following a positive test. After thorough instructions, the participants performed the tests at home and sent a digital photograph of each test to the study manager for confirmation. For cycle length estimation, women also provided information on the menstrual onset for the test and the subsequent cycle.

During each of the 10 test sessions, participants also collected a saliva sample via passive drool (1.5–2 ml of saliva in SaliCaps, IBL International, Hamburg, Germany) following established protocols [34]. When samples showed visible signs of contamination (e.g. blood) participants were asked to repeat the sample. Saliva samples were labelled and stored at −80°C within 10 min of sample collection until they were shipped on dry ice to the Kirschbaum Lab (Technical University of Dresden, Germany), where they were stored at −20°C until analysis. After thawing, the saliva was centrifuged at 3000 r.p.m. for 5 min to obtain a clear supernatant of low viscosity. Oestradiol and progesterone concentrations were measured using commercially available chemiluminescence immunoassays with high sensitivity (IBL International, Hamburg, Germany). Mean levels were 4.27 pg/ml for oestradiol (s.d. = 2.1, range = 1.57–21.39 pg/ml) and 39.63 pg/ml for progesterone (s.d. = 29.44, range = 11.31–176.63 pg/ml). The intra- and inter-assay coefficients were below 9% for both steroid hormones, which is considered good [36].

We assigned the estimated day of ovulation for each woman as the day after her first positive LH test. For women who had positive tests on two consecutive days (n = 11), the estimated day of ovulation was still assigned as the day after the first positive test. After validating that the estimated ovulation date was consistent with the expected range of the luteal phase [37], we plotted the 10 salivary hormone values relative to ovulation for each participant to assess whether the day of ovulation, as determined by the preceding steps, visually aligned with the overall hormonal pattern. Owing to the sampling density around the fertile window, we particularly relied on visually assessing oestradiol. We observed a pre-ovulation increase in oestradiol levels for each participant. For women with a sufficient number of post-ovulation hormone values, we also visually examined whether there was a discernible declining trend in oestradiol levels. We then assigned corresponding conception risk estimates (cf. [34], electronic supplementary material, S3.2). The cycles of all 29 women were regular (all cycles: mean = 30.48 days, s.d. = 3.31, range = 25–35). Positive LH tests were observed between reverse cycle days—18 and 13—and occurred on average on reverse cycle day—14.54 (s.d. = 1.2). One woman showed only negative LH tests as well as inconclusive hormonal patterns, most probably indicating a non-ovulatory cycle, and was therefore excluded from further analysis.

### (d) Statistical analysis

All statistical analyses were performed using R v. 4.1.3 [38]. The threshold for statistically significant *p*-values was set at 0.05 in all analyses.

#### (i) Test predictors

Statistical analysis for both perceptual and chemical analysis was conducted using the same test predictors: conception risk, oestradiol and progesterone values and the temporal variable ‘distance to ovulation’, all obtained for each sampling day (electronic supplementary material, table S4.1).

As the optimal transformation for cyclic hormones in linear mixed models is still under debate, both *z*- and log-transformations were applied. For *z*-transformation, subject-mean centring of hormone levels was followed by *z*-scaling (cf. [34]). To address negative values arising from subject-mean centring, log-transformation was performed before the centring (cf. [39]). We ran statistical analysis with either transformation but otherwise identical procedure, to rule out that the choice of transformation affected model results. We report the results based on *z*-transformation in the main text and details on the log-transformation in electronic supplementary material, S7.2 and S9.2.

## 3. Male odour perception

### (a) Odour collection procedure

For each session, odour donors were provided with odourless cotton gauze pads (10 cm × 10 cm) in pre-cleaned 30 ml amber glass vials, disposable gloves and medical adhesive tape (electronic supplementary material, table S5.1) as well as written instructions on how to apply the cotton pads (electronic supplementary material, S5.1). Women affixed the cotton pads directly onto the skin under both armpits using the provided tape, wore them for 12 h overnight prior to each sampling session, and removed their pads at the lab the next day. The samples were placed in clean glass vials, sealed using airproof polytetrafluoroethylene tape (electronic supplementary material, table S5.1), and stored at −80°C within 10 min of sample collection [40]. Participants wore their pads on average 12.68 h (s.d. = 1.92, range = 6–20). In total, test sessions were omitted five times across all women, thus we were able to collect 285 out of the 290 anticipated axillary cotton pads. Pads that were not returned properly (*n* = 2) and all odour samples of one participant showing no indication of ovulation (*n* = 10) were excluded. The remaining 273 axillary samples were evaluated in the corresponding rating sessions.

### (b) Odour raters

Heterosexual healthy men between 18 and 40 years old with normal olfaction were eligible as odour raters (electronic supplementary material S6.1). Men were recruited via the same online participant database as recruited women, bulletins, private contacts and social media. A total of 91 men (aged 19–40 years, mean = 25.47, s.d. = 4.57) participated as raters, of which 43 were in a romantic relationship, 44 were single and 4 did not indicate their relationship status.

### (c) Odour rating design and procedure

We decided to present samples from the left armpit only, as no laterality-related perceptual differences were shown for axillary odour [41]. To ensure that men would not smell the same women twice, a given man rated the odour of a total of 24 different women in two corresponding sessions over a 2 week interval (session A: 10 samples and olfactory performance test, session B: 14 samples). After rating half of the samples per session, raters were given a 15 min break to prevent immediate sensory exhaustion. Odour sample presentation was completely randomized with regard to odour donor ID and sample ID (i.e. the day it was collected within the cycle). Raters’ general olfactory ability was validated using the Sniffin’ Sticks test (Screening 12 Test; Burghart Messtechnik GmbH, Holm, Germany).

Participants received detailed written information about the procedure and inclusion criteria as well as the behavioural restrictions and infection protection measures applied. To avoid influences on olfactory sensitivity, odour raters were asked to refrain from consuming alcohol or any other recreational drug 12 h before and to not eat or drink (except water) 1 h before each session. Raters were told that they would have to evaluate axillary odour from different women.

Odour samples were removed from the freezer 2 h prior to each rating session and left to defrost at room temperature. Samples were presented in the opaque glass vials they had been collected and stored in. Each rater was assigned a permanent desk at which they received written instructions about the smelling and rating procedure (electronic supplementary material, S6.2), alongside the first odour sample to be rated. The sample order for each rater was predefined, and the samples were rotated clockwise between the desks until all raters evaluated every sample in the room. Raters were asked not to communicate their ratings. Raters wore cotton gloves and were instructed not to exhale on the samples to avoid contamination of the samples and glass vials. Smelling frequency per sample was not restricted; however, a maximum of two sniffs was recommended. Men gave their ratings immediately after smelling each sample on a paper questionnaire using verbally anchored 11-point Likert scales for the dimensions, ‘attractiveness’, ‘pleasantness’ and ‘intensity’, from −5 (*extremely unattractive/unpleasant/weak*) to +5 (*extremely attractive/pleasant/intense*) (electronic supplementary material S6.3). Raters were able to select ‘I cannot smell this sample’ if they found any sample too subtle to assess. Finally, demographic data and raters’ compliance with the behavioural restrictions were collected (electronic supplementary material, S6.3). Raters were supervised by male experimenters [42]. The male experimenters were blinded to the odour sample ID and corresponding fertility condition. Odour samples were sealed and frozen at −80°C immediately after each rating session until the next use (defrost frequency: mean = 1.91, s.d. = 1.06, range = 1–5). Overall, axillary samples were rated on average 7 times (s.d. = 2.79, range = 2–12) by a total of 91 men, of whom 10 only completed the first rating session. In total, 1938 ratings were completed, of which 152 (5.2 %) were rated as non-perceivable. Individual agreement inter-rater reliabilities (ICC 1,1) were ICC = 0.40 for intensity, ICC = 0.22 for attractiveness and ICC = 0.28 for pleasantness, which is in the expected range for body odour ratings [43].

### (d) Statistical analysis

To assess the relationship between female fertility and the three rating dimensions, *attractiveness*, *pleasantness* and *intensity*, we computed cumulative link mixed models (CLMMs), which are linear mixed models specifically designed for ordinal response data, such as Likert scale ratings [12,44]. As such, CLMMs retain the advantages of (G)LMMs in terms of accounting for random effects, accommodating correlated data and allowing for the inclusion of fixed-effect predictors. We conducted separate analyses for each dimension using the function *clmm* of the R package ‘ordinal’ (v. 2020-08-22 [45]). All ratings marked as perceivable were entered into the respective models. To assess the effects of each test predictor (electronic supplementary material, table S4.1) while accounting for variance explained by other predictors, we also included the following control variables into the models: the storage duration of the odour sample, the defrost frequency of the odour sample at the time of rating, the duration the pad was worn by the odour donor and the age of odour donors and raters. As random effects, we added odour donor ID, odour rater ID, odour sample ID and rating session ID (electronic supplementary material, table S7.2). Details of the model fit and checks are reported in electronic supplementary material, S7.1.

To assess the overall significance of the test predictors and to avoid ‘cryptic multiple testing’ [46], we compared the fit of each full model with a null model lacking the effects of the test predictors, but still containing all other terms using a likelihood ratio test (LRT) [47]. Only when the full-null model comparison was significant we proceeded with testing the significance of the individual predictors using LRTs.

Most prior studies, in contrast to ours, have focused on testing individual test predictors. To facilitate comparisons with these studies, we further assessed conception risk as the sole test predictor (excluding hormonal predictors and distance to ovulation) and conducted a parallel analysis using oestradiol and progesterone as the sole test predictors (excluding conception risk and distance to ovulation), each with an otherwise identical model structure (electronic supplementary material, S7.1). These analyses were performed for axillary attractiveness and pleasantness, as their full-null model comparison was significant in our original analysis. Additionally, the temporal variable ‘distance to ovulation’ is a novel addition compared with variables used in prior studies. Thus, in an exploratory manner, we assessed all of our original analyses without this variable to facilitate comparison with prior studies. We also explored the association between mean ovarian hormone levels and mean odour attractiveness across women (electronic supplementary material, S7.1). We also assessed whether violations of participants to sampling (*n* = 19) and rating (*n* = 4) restrictions affected scent ratings in robustness analyses, omitting cases with these violations. Additionally, we conducted robustness analyses to confirm that none of our test predictors had a general effect on the perceptibility of odour samples. Corresponding details are reported in electronic supplementary material, S7.1.

### (e) Results

**Figure 2.**
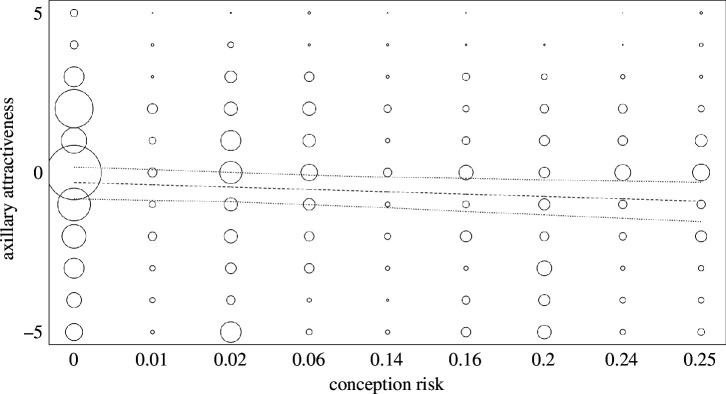
Ratings for axillary attractiveness predicted by conception risk. Ratings on the *y*-axis range from *extremely unattractive* (−5) to *extremely attractive* (+5). Bubbles represent the relative rating frequency, while the bubble area corresponds to the number of ratings (ranging from 2 to 12). The dashed line shows the model line while the dotted lines depict the bootstrapped 95% confidence intervals.

Men’s odour ratings were not compellingly linked to female fertility. Full-null model comparisons were significant for attractiveness and pleasantness, but not for intensity (LRT, attractiveness: χ^2^ = 9.81, d.f. = 4, *p* = 0.044; pleasantness: χ^2^ = 10.75, d.f. = 4, *p* = 0.030; intensity: χ^2^ = 2.02, d.f. = 4, *p* = 0.733). However, no statistically significant effect was found for the individual test predictors. In contrast to our predictions, axillary odour was descriptively evaluated as both less attractive and less pleasant at higher conception risk, but robustness analyses pointed towards inconsistent results (attractiveness: χ^2^ = 3.81, d.f. = 1, *p* = 0.051; pleasantness: χ^2^ = 3.05, d.f. = 1, *p* = 0.081, figure 2 and table 1). The magnitude of difference in the fitted average ratings from the lowest to the highest value of conception risk was 0.59 scale points for attractiveness and 0.49 for pleasantness. Models fitted with log-transformed hormones as well as robustness analyses support these results (electronic supplementary material, S7.2). Conception risk showed a significant effect on axillary attractiveness and pleasantness both when tested individually and in models excluding the variable ‘distance to ovulation’. However, contrary to our expectations, its estimate was negative, and the magnitude of difference low, both aligning with our initial analysis (see electronic supplementary material, S7.2). The results for oestradiol and progesterone, tested individually, as well as for axillary intensity without distance to ovulation, were consistent with those in the main analysis and are reported in electronic supplementary material, S7.2. We found no significant correlation for mean oestradiol or mean progesterone and mean odour attractiveness between women (oestradiol: ρ = 0.32, *p* = 0.118, progesterone: ρ = 0.06, *p* = 0.767, see electronic supplementary material, S7.2). Details on male ratings relative to ovulation and ovarian hormone levels are presented in electronic supplementary material, figures S7.1 and S7.2.

**Table 1.** Model results of male attractiveness and pleasantness ratings of women’s axillary body odour for test and control predictors (log odds, together with standard errors, confidence limits and significance tests, all d.f. = 1).

	**attractiveness**						**pleasantness**					
	log odd^(a)^	SE	5% CI	95% CI	χ2	*P*	log odd^(a)^	SE	5% CI	95% CI	χ2	*P*
**predictors**												
conception risk	−2.11	1.05	−4.21	−0.11	3.81	0.051	−1.83	1.04	−3.85	0.32	3.05	0.081
oestradiol^(b)^	0.06	0.07	−0.07	0.17	0.77	0.381	0.05	0.07	−0.08	0.19	0.53	0.465
progesterone^(b)^	0.13	0.07	−0.01	0.27	3.15	0.076	0.13	0.07	−0.01	0.27	3.18	0.075
distance to ovulation^(c)^	0.01	0.16	−0.30	0.32	0.03	0.858	0.01	0.15	−0.31	0.33	0.00	0.949
male age^(d)^	−0.11	0.14	−0.39	0.15	0.61	0.437	−0.14	0.12	−0.39	0.09	1.36	0.244
female age^(d)^	−0.06	0.19	−0.43	0.34	0.09	0.761	0.05	0.12	−0.36	0.45	0.08	0.785
storage duration^(d)^	0.09	0.17	−0.23	0.39	0.31	0.579	0.20	0.16	−0.09	0.51	1.61	0.204
number defrosted^(d)^	−0.09	0.14	−0.38	0.16	0.37	0.543	−0.09	0.13	−0.34	0.17	0.53	0.469
pad wear duration^(d)^	−0.11	0.08	−0.27	0.04	1.95	0.163	−0.15	0.09	−0.32	0.03	2.83	0.092

^a^ Log odds represent the logarithm of the odds ratio for an individual moving up one rating scale point. Log odds are comparable to the estimates of linear mixed models.

^b^ Subject-mean-centred and *z*-transformed to a mean of 0 and a s.d. of 1.

^c^ log-transformed (base e).

^d^ *z*-transformed to a mean of 0 and an s.d. of 1, see electronic supplementary material, table S7.2 for mean and s.d. of the original variables.

## 4. Chemical study

The chemical samples were collected in parallel to the rating samples in 16 of the 29 women, ensuring that the procedures for fertility measures and general odour collection were consistent. Our chemical analysis relied on chemical substances present in women’s axillary odour, which easily evaporate at room temperature and can be directly detected by the main olfactory epithelium of the receiver [48] (i.e. semi-volatiles with boiling points between 250 and 400°C and volatiles with boiling points below 250°C [49]).

### (a) Chemical sample collection

Chemical odour samples were collected by drawing the ambient air around women’s armpits into thermal desorption (TD) tubes (stainless steel TD tubes, 0.25 in. × 3.50 in., Supelco, Bellefonte, USA) via a portable, two-channel air pump (BiVOC2, Umweltanalytik Holbach GmbH, Germany). The compounds present in the odour sample are adsorbed onto polymers inside the tube, enabling storage for subsequent chemical analysis [50]. For each sample, 0.5 l of air (flow rate: approx. 1.5 l/min; [51]) was collected. For chemical analyses, TD samples are more suitable than cotton swab samples, but cannot be applied for testing odour perception, as the collected compounds are depleted during the analysis. However, there is a substantial overlap between the compounds collected using TD tubes and those using cotton pads applied in the perception tests [50]. This combined approach, leveraging the strengths of both methods for their respective applications, has already been successfully applied to demonstrate olfactory fertility cues in a non-human primate including chemical analysis and perception tests with male conspecifics [52]. Women performed odour collection alone at the lab. In practice trials, they were thoroughly instructed on how to handle the equipment and properly position the tube approximately 3 cm above the left armpit. Participants were handed a new TD tube if their previous tube came into contact with skin, or was dropped on the ground, to avoid contamination. Between sessions, the test room was ventilated for 60 min to establish neutral odour conditions. Additionally, two types of TD tube blanks were collected and analysed: analytical blanks (i.e. similar handling and processing as all other samples, but no air was pulled through these tubes, *n* = 27) and room blanks (i.e. samples with air of the testing room at least 60 min before or after participants were present, *n* = 12) to distinguish human compounds from external contamination. Two participants omitted one test session each, resulting in 158 chemical odour samples being collected.

### (b) Chemical analysis

We used GC–MS to measure the composition of the chemical odour sample. For each sample, we obtained a corresponding chromatogram, in which the containing peaks indicate the presence and intensity of individual chemical compounds present in women’s axillary odour.

GC–MS measurement failed for 15 samples, leaving a total of 143 samples for further data analysis. For each sample, we obtained a corresponding chromatogram with peaks that provide information about the components present, their relative concentrations and retention times (i.e. duration until separation from the other components). We first determined recurring peaks across all chemical samples to identify compounds that likely originated from axillary odour. Initially, we identified 118 recurring peaks. To eliminate substances of non-human origin (e.g. from sampling material), we discarded peaks from substances known to be contaminants from previous studies using the same TD tubes and analytical methods (*n* = 10 [51]), and removed compounds that exhibited equal or higher intensities (i.e. peak areas) in blank samples than in human samples (*n* = 5), leaving 103 compounds for statistical analysis. In electronic supplementary material, S8, we have provided detailed technical information on the chemical analysis.

### (c) Statistical analysis

Female fertility was tested with regard to: (i) the overall similarity of whole body odour profiles [53,54] and (ii) compound composition (i.e. the presence and relative intensity of specific compounds) between chemical profiles [52,53] based on 143 axillary odour samples of 16 women.

#### (i) Similarity between odour profiles

We tested how female fertility affects the overall similarity of whole body odour profiles by computing pairwise Bray–Curtis indices calculated from standardized, log(*x* + 1)-transformed peak areas [54]. This index is computed by comparing the relative intensities of all compounds between pairs of samples, resulting in a single value quantifying the similarity of two chemical profiles. Similarity indices between all pairs of samples were related to similarities in test predictors with permutational multivariate analysis of variance with distance matrices (ADONIS) using the function *adonis2* of the R package ‘vegan’ (v. 2.6-2 [55]). All four test predictors were entered in a single ADONIS. Hence, we determined whether these predictors have a statistically significant effect on the overall similarity or dissimilarity of body odour profiles.

#### (ii) Fertility information and chemical composition

Within individual odour samples, the intensities of specific components relative to the overall composition of that sample may provide fertility-related information. Alternatively, fertility information may be discernible through variations in composition across different odour samples. To address this, we conducted statistical analyses using two different data transformations on compound intensity (i.e. peak area).

First, we applied normalized relative peak areas per compound, centred to a mean of zero and scaled to a s.d. of one, in which all compounds are adjusted to the same size, giving them the same importance in the analysis. This allows investigating changes in the relative abundance of compounds *between* samples. Second, our response was log(*x* + 0.01) and arcsine transformed, which translates into relative peak areas per compound. Log-transformation compensates for the relative impact of very abundant compounds, enabling the analysis of relative differences in the abundance of compounds *within* the same odour sample.

We assessed the relationship between female fertility and axillary chemical composition by fitting linear mixed models (LMM) using the function lme of the package ‘lme4’ (v. 1.1-28 [56]). All models were fitted with a vectorized multivariate data matrix of odour samples (*n* = 143) and compounds (*n* = 103), with relative peak areas (143 × 103 = 14 729) as the response variable. Control predictors were the duration the respective TD sample had been stored before the GC–MS analysis (in months), the GC–MS batch the tubes were analysed in and the age of odour donors. Samples (matrix rows) and compounds (matrix columns) were included as random intercepts to avoid pseudoreplication and heteroscedastic variance [57]. Odour donor ID was fitted as an additional random factor (see electronic supplementary material, table S9.2). Importantly, compounds most strongly associated with female fertility can statistically be identified based on the steepness of their slope estimates (i.e. showing the steepest slopes [53]). Thus, we fitted the test predictors as random slopes within the random effect compound, which acted as our actual test predictors in these models. Additionally, all biologically possible and meaningful random slopes and interactions were fitted to obtain more reliable *p*-values [58]. Details of the model fit and checks are reported in electronic supplementary material, S9.1. Again, we conducted full-null model comparison using LRT. Additionally, for the chemical analysis, we conducted exploratory statistical analyses without the variable ‘distance to ovulation’. We also assessed whether violations of odour donors to sampling restrictions affected the scent composition in robustness analyses. All are reported in electronic supplementary material, S9.1.

### (d) Results

We detected no relationship between the overall similarity of women’s odour profiles and the test predictors (ADONIS, *r* = 0.32, *p* = 0.711), suggesting that the similarity between odour profiles was not related to the similarity between test predictor values.

We also found no significant effect of female fertility predicting women’s axillary odour composition for the log-transformed (LRT, χ^2^ = 15.49, d.f. = 22, *p* = 0.841) or the normalized response (LRT, χ^2^ = 27.29, d.f. = 22, *p* = 0.201). Models with log-transformed hormones confirm these results and robustness analysis showed that these patterns were not significantly affected by external contamination of odour samples (electronic supplementary material, S9.2). When tested without ‘distance to ovulation’, results were consistent with our original analyses (electronic supplementary material S9.2).

## 5. Discussion

We provide a combined analysis of chemical and perceptual shifts in the axillary odour of women in relation to female fertility based on frequent and hormonally confirmed odour samples from the same women. Our results revealed no evidence that men’s attraction to women’s body odour is positively predicted by female fertility. These results are corroborated by the lack of cycle-related variation in the volatile composition of axillary odour, as suggested by our chemical analyses.

We found no compelling indication that men’s perception of female axillary odours varies with female fertility. Although the full-null model comparison including all test predictors showed significance, none of the test predictors had a robust effect on how attractive and pleasant men perceive women’s axillary odour in a single encounter (table 1). Conception risk was significantly associated with attractiveness and pleasantness when models were analysed without ‘distance to ovulation’ as well as when modelled as the sole test predictor (to account for any potential statistical mediating effects of reproductive hormones in our models). Here, *p*-values fluctuated more or less closely around the statistical level of significance, particularly for axillary pleasantness (electronic supplementary material, tables S7.7, S7.8 and S7.10). Importantly, however, in all models, differences in average ratings between the lowest and highest conception risk values were very small and in the opposite direction of what we expected. Thus, our results do not provide evidence for a positive effect of conception risk on men’s perception of female axillary odours. In several previous studies using *repeated* encounters with the same woman, male attractiveness and pleasantness ratings were higher for female odour of high fertility [8,20,21]. Hence, male attraction to a woman’s axillary odour in a *single* encounter seems to be little affected by her current fertile state, if this effect is present at all. Fertility-related cues in women’s body odour, as well as men’s ability to detect them, have already been suggested to be less robust than previously assumed, based on evidence from studies with more rigorous methods [22,23]. In the most recent work of Mei *et al*. [22], male attraction to women’s axillary odour was assessed based on five to six hormonally confirmed odour samples across an entire cycle. Although they found significant mean attractiveness rating effects, men’s detection ability of female fertile state from scent attractiveness was low, which our results support. In our study, we further found no compelling evidence that cyclic hormones, conception risk or temporal distance to ovulation were associated with men’s perception of axillary intensity, even when accounting for sampling violations. Evidence for fertility-related variation in axillary intensity is mixed and includes null effects [21,59]. Even in studies reporting significant effects [8,20], cyclic variations in axillary intensity were statistically less pronounced than those of attractiveness or pleasantness ratings.

Cycle-related fluctuations in oestradiol and progesterone did not seem to be linked to within-woman shifts in male odour ratings, regardless of the rating dimension. Another study that has investigated this relationship also could not find a link between ovarian hormone levels and intra-individual shifts in odour attractiveness [22], which is in line with our results. Thus, we still lack direct evidence of hormonal correlates explaining the elevated mid-cycle odour attractiveness ratings reported in previous studies (e.g. [8,20,21]). Further studies are required to investigate this in greater depth.

Solely considering the perceptual aspect of odour attractivity lacks the crucial understanding of whether and how chemical information about female fertility might manifest in body odour. Fertility-related substances could change independently of the rest of the chemical profile over the cycle, which would need to be tracked over time to decode its fertility information (tested in the normalized model). Alternatively, fertility-related substances could change relative to the overall profile, resulting in chemical profiles being specific for certain fertility information that is immediately interpretable from its composition without tracking it over the entire cycle (tested in the log-transformed model). However, in our study, we found no convincing evidence that shifts in female fertility are associated with chemical variation in the composition of women’s axillary odour in either type of model, substantiating our results from the perceptual study. Furthermore, the similarity between the whole chemical profiles was not significantly linked to female fertility information. The results were independent of the type of hormone level transformation and remained robust when controlling for external contamination stemming from odour donor violations.

It is still unknown how cyclic fluctuations in conception risk and ovarian hormone levels might translate into chemical body odour variation. This may occur with a time lag that prevents chemical variation from accurately reflecting variation in female fertility. Thus, female axillary odour may not contain fertility information that is usable for men or is at least not salient enough to be decoded. Initial sexual encounters do not necessarily result in the formation of pair bonds [60], an important factor for promoting offspring survival in humans (reviewed in [61]). Furthermore, single encounters do not provide a high degree of familiarity, which has been suggested to affect how pleasant and intense humans evaluate odours [62] but see [63]. Some of the earlier studies showing cyclic shifts in odour attractiveness presented men with multiple samples of the same woman [8,20]. Thus, multiple encounters with different odours of the same woman could help men to better distinguish potential olfactory fertility cues as they gain higher familiarity with her scent [26]. However, in a more recent study, men were very poor at discriminating the female’s fertile state from odour attractiveness even with multiple odour samples of the same women in direct comparison [22]. As a promising future directive, olfactory fertility cues could be investigated in more depth in scenarios mimicking different levels of familiarity and commitment between the partners, as mating motivation could be shaped, e.g. by the desire to have children. It would be interesting to see whether these factors have any effect on female body odour composition or male olfactory perception of their partner.

Both human body odour formation and the ovulatory cycle involve intricate physiological processes, which make it difficult to fully address this complex interplay methodologically. Our sampling design covered the entire ovulatory cycle but particularly focused on the days around ovulation to be able to test also for gradual and potentially very subtle variations during the fertile window. Our analytical approach allowed us to statistically relate odour composition or ratings to test predictors over the entire cycle despite the focus on the fertile window, and luteal progesterone was reasonably well covered across the participants. However, with only one luteal sample per woman, we may not have captured their luteal progesterone maximum. Hence, more samples outside the fertile window would have been helpful to cover the full range of progesterone values and improve the accuracy of the hormonal profiles. Similarly, although the 10 saliva samples per woman and cycle provide consistent results, multiple daily samples and several cycles per woman would offer more stable hormonal profiles [30]. Furthermore, most conventional immunoassays were shown to exhibit limited validity for assessing salivary oestradiol concentrations, and thus, should be interpreted with caution [64]. However, assay validity does not fully explain our results being statistically non-significant, as we also did not detect any compelling effects for conception risk or distance to ovulation.

Similar to most other studies investigating cyclic shifts in body odour, we provided no specific instructions on shaving axillary hair. In a previous study [65], only weak effects of axillary hair on body odour perception were found, and we are not aware of studies assessing the impact of axillary hair on the chemical body odour composition. Furthermore, we statistically controlled for odour donor ID, but the potential influence of axillary hair may not have been fully addressed. On the other hand, we standardized various other aspects of odour collection (i.e. diet, behaviour and personal hygiene) to eliminate factors suggested to enhance or mask aspects of a person’s body odour and thus mediate its perception [66]. For example, our odour donors followed a vegan/vegetarian diet, potentially influencing our results differently from studies without dietary restrictions. Moreover, while a standardised laboratory setting, such as the one we used, allows presenting olfactory stimuli with minimal external distortion, it may not fully resemble how odours would be perceived in a naturalistic setting. While controlled laboratory studies are the standard in social olfaction research, whole body odour in a live interaction was found to provide more social information than samples from an isolated odour source such as the armpit [67]. This would also be a promising approach to further investigate the dynamic interaction between odour and human mate choice.

Overall, when comparing methodologies and analytical approaches across studies, the field remains highly heterogeneous, which may to some extent explain differences in results. Consequently, further studies are needed (simulating, e.g. both single and repeated encounters) to assess what might account for the different magnitudes and directions of the observed effects. It will be particularly important to understand whether these differences arise from methodological aspects and/or if different studies also capture biologically distinct aspects. More studies are needed that combine perceptual and chemical analyses, hormonally confirmed measures of ovulation and state-of-the-art analytical approaches with robust sample sizes to assess which results are replicable and to understand the dynamic processes that shape olfactory fertility cues and their perception.

Together, we provide a successful application of combined perceptual and chemical data to investigate fertility-dependent shifts in women’s body odour and highly encourage advancing our study to determine whether our findings replicate.

## 6. Conclusion

Using frequent odour samples from the same women and hormonal assessment of fertility, we assessed potential fertility-related shifts in axillary body odour in a twofold study combining perceptual and chemical evidence. Overall, there was no compelling evidence that female fertility positively affects male odour ratings, aligning with prior highly powered studies questioning men’s ability to detect cycle-related shifts in women’s body odour attractiveness. We also did not find direct hormonal evidence explaining the elevated mid-cycle odour attractiveness ratings reported in previous studies. Furthermore, the chemical composition of a woman’s axillary odour was not affected by her current fertile state or fluctuating ovarian hormone levels. We are certainly still at the beginning of understanding the physiological interaction between the gradual fluctuations in fertility and ovarian hormones across the ovulatory cycle and women’s body odour. Nevertheless, this raises the possibility that research on cyclic shifts in women’s body odour might have to be revisited, as we would need to establish whether or not there are fertility-related correlates in female body odour in the first place. We strongly encourage further disentangling the physiological basis as well as the social function of olfactory cues to female fertility in humans with the robust methods we have at hand. It would also be intriguing to see this line of research transferred into a more naturalistic setting to offer a greater ecological validity of this paradigm.

## Data Availability

All data are available online at the Open Science Framework under the following link [68]. Supplementary material is available online [69].
